# Living space, utilities, and communication access as determinants of intrinsic capacity: longitudinal findings from England and China

**DOI:** 10.1186/s12877-026-07549-w

**Published:** 2026-04-24

**Authors:** Eric TC Lai, Ziting Huang, Jean Woo

**Affiliations:** 1 Department of Medicine & Therapeutics, Faculty of Medicine, The Chinese University of Hong Kong, Shatin, Hong Kong SAR China; 2 Institute of Health Equity, The Chinese University of Hong Kong, Shatin, Hong Kong SAR China; 3 Jockey Club Institute of Ageing, The Chinese University of Hong Kong, Shatin, Hong Kong SAR China

**Keywords:** Intrinsic capacity, Healthy ageing, Housing characteristics, Hross-national study

## Abstract

**Background:**

Housing is a critical component of age-friendly environments and may influence intrinsic capacity (IC), a multidimensional indicator of healthy ageing defined by the World Health Organization. However, longitudinal evidence on how housing characteristics relate to IC across diverse contexts remains limited.

**Methods:**

Data were collected from the China Health and Retirement Longitudinal Study (CHARLS) (*N* = 5,691) and the English Longitudinal Study of Ageing (ELSA) (*N* = 5,218). IC was measured across five domains including cognition, locomotor, vitality, sensory, and psychological well-being. Housing characteristics covered living space, basic utilities and infrastructure, communication access, housing problems, and accessibility/adaptation features. The relationship between housing characteristics and IC was assessed using multivariable linear mixed-effects growth-curve models adjusted for socio-demographic and health-related covariates. Sensitivity analyses additionally modelled post-baseline IC while adjusting for baseline IC and baseline covariates.

**Results:**

In the primary models, most housing characteristics were associated with baseline differences in IC, whereas evidence for differential IC trajectories over follow-up was limited. In CHARLS, greater living space, utility access, and communication access were associated with higher baseline IC. In ELSA, fewer housing indicators were associated with baseline IC, and computer ownership was the only housing characteristic associated with a more favourable IC trajectory in the primary models; this association attenuated after adjustment for baseline IC. In the baseline-IC adjusted sensitivity analyses, home adaptation in ELSA was associated with a less favourable IC trajectory.

**Conclusions:**

Housing characteristics were associated primarily with IC level differences, with limited evidence of differential IC decline over follow-up. Adequate living space and communication access were consistently related to better IC levels, while utility-related associations were more evident in CHARLS than in ELSA. These findings support the relevance of housing within healthy-ageing policy, while also indicating that the meaning and implications of specific housing characteristics differ across settings and should be interpreted cautiously.

**Supplementary Information:**

The online version contains supplementary material available at 10.1186/s12877-026-07549-w.

## Introduction

The world’s population is ageing rapidly, creating an urgent need to ensure that longer lives are also healthier, more independent, and more meaningful [1, 2]. According to the World Health Organization (WHO) healthy ageing framework, healthy ageing is defined not as the absence of disease, but as the process of developing and maintaining the functional ability that enables older people to do the things they have reason to value [3]. Central to this framework is intrinsic capacity (IC), defined as the composite of an individual’s physical and mental capacities [4]. IC is commonly conceptualised across five domains—locomotion, cognition, vitality, sensory capacity, and psychological well-being. Intrinsic capacity is a dynamic attribute that can change across the lifespan and is a powerful predictor of an individual’s health trajectory [5], because a decline in intrinsic capacity often precedes the onset of significant functional limitations and dependence. Lower IC has been shown to be related to with frailty, disability, care dependency, and mortality [6].

The environments in which older people live are important determinants of their health and well-being [7]. Housing is especially relevant in later life because older adults often spend a large proportion of their time at home, and the home environment may either support or constrain daily functioning [8, 9]. Previous research has shown that poor housing quality—such as inadequate heating, dampness, poor ventilation, and structural disrepair—is associated with poorer physical and mental health [10]. Housing accessibility and design are also important: hazards such as stairs without railings, slippery surfaces, or inaccessible toilets may increase falls risk and limit independence, whereas adaptations and supportive design may help people remain active and age in place [11].

Although these links between housing and health are well established, much less is known about how housing characteristics relate specifically to IC as a multidimensional indicator of healthy ageing [12]. This is an important gap because housing characteristics may influence IC through multiple and overlapping pathways, rather than affecting only one domain at a time. For example, adequate living space may support locomotion through safer movement indoors, reduce crowding-related stress and psychological burden, and facilitate social interaction that benefits cognition and psychological well-being [13–15]. Heating and other basic utilities may influence vitality and psychological health by reducing thermal stress and improving everyday living conditions [7, 16]. Indoor toilets and related accessibility features may support locomotion, reduce fall risk, and preserve independence [17, 18]. Communication technologies may also operate across several domains by supporting social connection, access to information and services, and coping with functional decline [19]. Accordingly, housing characteristics are best understood as multidimensional environmental exposures that may shape several IC domains simultaneously, rather than acting on only one domain [12].

Cross-national comparison may help identify both shared and context-specific housing–IC relationships [20]. The English Longitudinal Study of Ageing (ELSA) [21] and the China Health and Retirement Longitudinal Study (CHARLS) [22] provide an opportunity to examine these questions in two distinct settings. England represents a high-income welfare context with relatively well-developed housing infrastructure, whereas China is undergoing rapid social and economic transition alongside population ageing [23]. These differences are analytically useful, but they also require caution: the same housing indicator may not carry the same meaning in the two settings, and any cross-country comparison must therefore be interpreted with attention to differences in infrastructure, climate, and socioeconomic context [23].

In this study, we examine the associations between housing characteristics and IC in older adults using longitudinal data from ELSA and CHARLS. We focus on housing features related to living space, basic utilities and infrastructure, communication access, housing problems, and accessibility/adaptation. By analysing data from two contrasting national contexts, we aim to identify both common and context-specific patterns in the relationship between housing and IC, while situating these findings within the WHO healthy ageing framework.

## Methods

### Study population

**Fig. 1:**
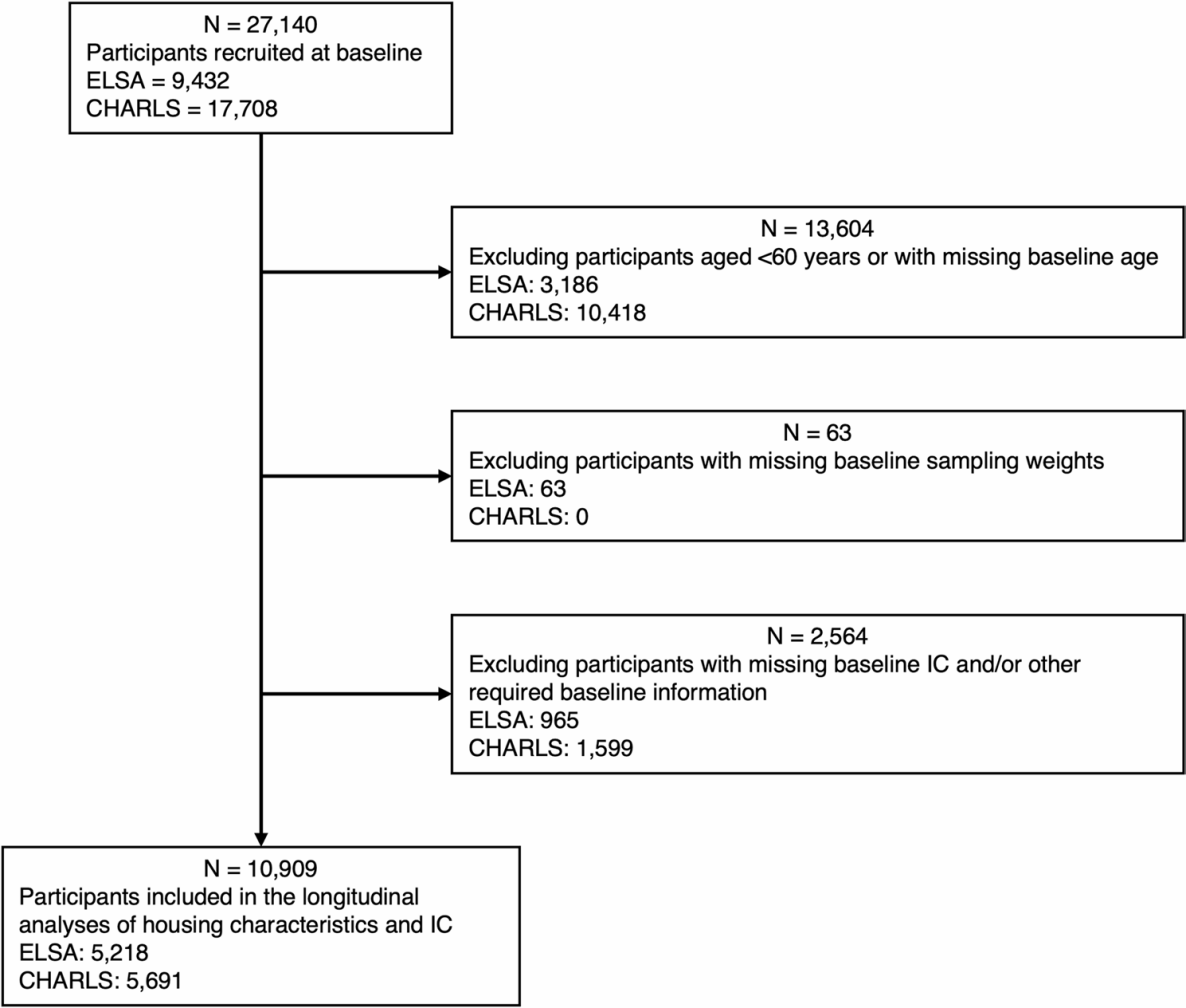
Flow chart of sample selection

The English Longitudinal Study of Ageing (ELSA) and the China Health and Retirement Longitudinal Study (CHARLS) are nationally representative studies designed to monitor the long-term health of community-dwelling older adults. Baseline interview took place respectively in 2004/05 and 2011/12 for ELSA and CHARLS. Cohort participants were followed up biennially thereafter. Given the availability of vitality data, for ELSA, we used data from follow up waves in year 2004/05, 2008/09, 2012/13 and 2016/17; for CHARLS, we used data from follow up waves in year 2011/12, 2013 and 2015. Participants were excluded if they were: (1) aged < 60 years at baseline, (2) had missing baseline sampling weights, or (3) had missing baseline information required for the housing analyses, including baseline IC and/or key housing and covariate measures. Figure 1 shows the inclusion/exclusion process.

### Intrinsic capacity

IC was conceptualised in accordance with the WHO healthy ageing framework as the composite of an individual’s physical and mental capacities across five domains: locomotion, cognition, vitality, sensory capacity, and psychological well-being [4]. The domain measures used in this study were harmonised across ELSA and CHARLS using available cohort items. Cognition was measured using immediate recall, delayed recall, and orientation in time. Psychological well-being was measured using Center for Epidemiologic Studies Depression Scale (CES-D) or European Depression Scale (EURO-D) for assessing depression. Sensory function was assessed using self-rated vision and hearing. Locomotion was measured using self-rated walking difficulty and chair-stand difficulty. Vitality was measured using grip strength and body mass index (BMI). Details of the specific items used to operationalise each domain are provided in Supplementary Table S1.

Because the distribution of IC derived from factor analysis and from a simple summative score has been shown to be similar, we summed the five domain scores (each scored 0–2) to generate an overall IC score ranging from 0 to 10 [24]. We used this overall score to reflect the WHO concept of IC as an integrated multidimensional construct rather than as five isolated domains [25]. A summative score is also straightforward to interpret and has been used in previous studies of IC measurement and scoring [26]. IC was therefore analysed as a continuous summary measure in the main models. This scoring approach for IC is straightforward to be implemented in clinical settings and easily understandable by different healthcare professionals; it also demonstrates good sensitivity and specificity [26]. Higher scores reflected better IC within each domain.

### Housing characteristics

The housing variables were classified as broad dimensions of the home environment for analytical clarity, while recognising that several of them may influence more than one IC domain. These dimensions included living space, basic utilities and infrastructure, communication-related resources, housing problems, and adaptation/accessibility features.

In ELSA, housing characteristics included home ownership, average number of rooms per person, heating, selected durable/communication-related items, property adaptations, and reported housing problems. In CHARLS, housing characteristics included home ownership, average number of rooms per person, heating, electricity, running water, gas supply, communication access (telephone and internet), and selected accessibility/sanitation items such as elevator, handicapped facilities, indoor toilet, and indoor toilet with a seat. Detailed coding and harmonisation procedures for the housing variables are provided in Supplementary Tables S2 and S3.

Adaptation-related variables (for example, ramps, handrails, lifts, elevators, and bathroom modifications) were included because they may represent either a supportive environment that enables functioning or, alternatively, a response to pre-existing functional decline. As such, the selected set of housing characteristics could be markers of both environmental support and possible underlying vulnerability.

### Covariates

All models adjusted for baseline age, sex, marital status, education, household wealth, and number of chronic conditions. These covariates were selected because they are plausible common causes of both housing conditions and IC and therefore potential confounders of the housing–IC association [27]. Chronic conditions included hypertension, diabetes, cancer, lung disease, heart problems, and stroke, and were grouped as 0, 1, or ≥ 2 conditions. Where required, missing baseline covariate were explicitly retained as a separate category for analysis and reporting.

### Statistical analysis

Baseline characteristics were summarised separately for ELSA and CHARLS. Continuous variables were described as mean (standard deviation), and categorical variables as number (percentage). Linear mixed-effects growth-curve models were used to investigate associations between each housing characteristic and overall IC over follow-up. Each housing characteristic was entered in a separate model together with time since baseline and a housing × time interaction term. Accordingly, the models estimated: (i) the annual change in IC in the reference group (i.e. the group without the housing characteristics of interest), (ii) the baseline IC difference between participants with and without the housing characteristic, and (iii) the difference in annual IC change associated with the housing characteristic. The primary models adjusted for the baseline covariates listed above.

We conducted the following sensitivity analyses. First, to evaluate possible attrition bias, we compared baseline characteristics of participants with complete versus incomplete IC follow-up. Second, we then estimated inverse-probability-of-inclusion weights based on baseline IC, socio-demographic characteristics, and housing measures, and refitted the mixed models using weighted mixed-effects models. We used *WeMix* in R programming, which is specifically designed to fit mixed-effects models with weights at multiple levels, building on multilevel pseudo maximum likelihood approaches for complex survey data [28]. Third, to address the baseline functional differences and possible reverse causality, we re-estimated the models using post-baseline IC observations only and additionally adjusted for baseline IC and baseline covariates. All analyses were conducted in R. Statistical significance was assessed using two-sided tests with *p* < 0.05.

### Ethics

This study used de-identified, publicly available data from the English Longitudinal Study of Ageing (ELSA) and the China Health and Retirement Longitudinal Study (CHARLS). All participants gave their consent to participate. Both studies obtained ethical approval and informed consent at original data collection (ELSA: National Research Ethics Service; CHARLS: Peking University Biomedical Ethics Review Committee, IRB00001052-11015). Access was granted through formal application processes, and all analyses complied with data use agreements. No additional ethical approval was required for this secondary analysis.

## Results

Figure 1 shows the inclusion and exclusion process for the revised analytic sample. A total of 5,218 participants were included in ELSA and 5,691 in CHARLS. In ELSA, 9,432 participants were included at baseline, of whom 3,186 were excluded because they were aged < 60 years or had missing baseline age, 63 were excluded because of missing baseline sampling weights, and 965 were excluded because of missing baseline intrinsic capacity (IC) and/or other required baseline information. In CHARLS, 17,708 participants were included at baseline, of whom 10,418 were excluded because they were aged < 60 years or had missing baseline age, and 1,599 were excluded because of missing baseline IC and/or other required baseline information.

Table 1 summarised the baseline characteristics of participants in CHARLS and ELSA. The mean age of the included participants in CHARLS and ELSA was respectively 67.2 and 70.7 years. The prevalence of several housing characteristics differed substantially across the two cohorts. For example, heating and at least one room per person were almost universal in ELSA but much less common in CHARLS. Intrinsic capacity and its component domains were also described at baseline for both cohorts. Marked cross-country differences in the prevalence of several housing characteristics were observed. For example, heating was reported by 95.0% of ELSA participants but only 9.2% of CHARLS participants, and at least one room per person was almost universal in ELSA (99.5%) but much less common in CHARLS (54.0%).

**Table 1:** Baseline characteristics of participants in CHARLS and ELSA

Characteristic	CHARLS (*N* = 5,691)	ELSA (*N* = 5,218)
Age	67.2 (6.0)	70.7 (7.6)
Intrinsic capacity (0–10)	8.07 (1.54)	8.14 (1.64)
Cognition (0–2)	1.72 (0.53)	1.65 (0.58)
Psychological (0–2)	1.53 (0.63)	1.51 (0.73)
Sensory (0–2)	1.56 (0.60)	1.91 (0.31)
Locomotion (0–2)	1.70 (0.50)	1.60 (0.64)
Vitality (0–2)	1.44 (0.62)	1.46 (0.63)
Sex		
Female	2719 (47.8%)	2876 (55.1%)
Male	2972 (52.2%)	2342 (44.9%)
Missing/Unknown	0 (0.0%)	0 (0.0%)
Marital status		
Married/partnered	4589 (80.6%)	3411 (65.4%)
Not married (single/divorced/widowed)	1102 (19.4%)	1806 (34.6%)
Missing/Unknown	0 (0.0%)	1 (0.0%)
Education		
Primary	5300 (93.1%)	2267 (43.4%)
Secondary	273 (4.8%)	1914 (36.7%)
Tertiary	118 (2.1%)	538 (10.3%)
Missing/Unknown	0 (0.0%)	499 (9.6%)
Total household wealth (tertiles)		
1	1585 (27.9%)	1639 (31.4%)
2	1330 (23.4%)	1720 (33.0%)
3	1299 (22.8%)	1804 (34.6%)
Missing/Unknown	1477 (26.0%)	55 (1.1%)
Number of chronic diseases		
0	2777 (48.8%)	1938 (37.1%)
1	1825 (32.1%)	1968 (37.7%)
≥2	996 (17.5%)	1312 (25.1%)
Missing/Unknown	93 (1.6%)	0 (0.0%)
Housing characteristics		
Home ownership	4589 (80.6%)	4285 (82.1%)
At least 1 room per person	3071 (54.0%)	5190 (99.5%)
Heating	521 (9.2%)	4957 (95.0%)
Electricity supply	4640 (81.5%)	
Running water	3459 (60.8%)	
Coal / natural gas supply	689 (12.1%)	
Telephone connection / phone ownership	2896 (50.9%)	5107 (97.9%)
Internet connection	588 (10.3%)	
Computer ownership		2337 (44.8%)
Any housing problem		1428 (27.4%)
Too cold in winter		146 (2.8%)
Any home adaptation		1467 (28.1%)
Elevator	4657 (81.8%)	
Handicapped facilities	4697 (82.5%)	
Indoor toilet	4105 (72.1%)	
Indoor toilet with seat	882 (15.5%)	

Values are mean (SD) for continuous variables and n (%) for categorical variables

The longitudinal model results are presented in Table 2. For clarity, we distinguish between three model components: the annual change in IC in the reference group (i.e. the group without the housing characteristic), the baseline IC difference between participants with and without each housing characteristic, and the slope difference per year associated with each housing characteristic. In the primary growth-curve models, most housing characteristics were associated with baseline differences in IC, whereas fewer were associated with differences in subsequent IC trajectories over follow-up. In both cohorts, the annual change in IC in the reference group was generally negative, indicating IC declining with age. In CHARLS, several housing characteristics were associated with higher baseline IC, but housing × time interaction terms were generally small and not statistically significant. In ELSA, a similar pattern was observed, with most housing indicators associated with baseline IC level rather than differential IC decline, except for computer ownership.

**Table 2:** Associations of housing characteristics with baseline intrinsic capacity and subsequent IC trajectories in CHARLS and ELSA

Cohort	Housing indicator	Annual change in IC in reference group	*p* (annual)	Baseline difference (Yes–No)	*p* (baseline)	Slope difference per year (Yes–No)	*p* (slope)
CHARLS	Home ownership	-0.104 (-0.135, -0.072)	< 0.001	-0.203 (-0.313, -0.092)	< 0.001	-0.006 (-0.042, 0.029)	0.719
CHARLS	≥ 1 room per person	-0.112 (-0.132, -0.092)	< 0.001	0.188 (0.104, 0.271)	< 0.001	0.006 (-0.022, 0.033)	0.691
CHARLS	Heating available	-0.112 (-0.126, -0.098)	< 0.001	0.326 (0.176, 0.477)	< 0.001	0.044 (-0.006, 0.094)	0.086
CHARLS	Electricity supply	-0.114 (-0.146, -0.082)	< 0.001	0.187 (0.079, 0.295)	< 0.001	0.006 (-0.029, 0.041)	0.736
CHARLS	Running water supply	-0.104 (-0.126, -0.082)	< 0.001	0.378 (0.291, 0.465)	< 0.001	-0.007 (-0.035, 0.021)	0.604
CHARLS	Gas supply	-0.113 (-0.127, -0.098)	< 0.001	0.331 (0.196, 0.466)	< 0.001	0.033 (-0.011, 0.077)	0.147
CHARLS	Telephone connection	-0.112 (-0.132, -0.092)	< 0.001	0.190 (0.106, 0.275)	< 0.001	0.006 (-0.021, 0.034)	0.653
CHARLS	Internet connection	-0.111 (-0.126, -0.097)	< 0.001	0.471 (0.328, 0.613)	< 0.001	0.028 (-0.019, 0.075)	0.239
CHARLS	Elevator	-0.101 (-0.134, -0.068)	< 0.001	-0.393 (-0.506, -0.279)	< 0.001	-0.010 (-0.046, 0.027)	0.607
CHARLS	Handicapped/access features	-0.117 (-0.152, -0.082)	< 0.001	-0.210 (-0.325, -0.095)	< 0.001	0.009 (-0.029, 0.047)	0.637
CHARLS	Indoor toilet	-0.106 (-0.131, -0.080)	< 0.001	0.192 (0.098, 0.286)	< 0.001	-0.005 (-0.035, 0.026)	0.763
CHARLS	Indoor toilet with seat	-0.111 (-0.125, -0.096)	< 0.001	0.466 (0.344, 0.588)	< 0.001	0.010 (-0.029, 0.049)	0.612
ELSA	Home ownership	-0.069 (-0.084, -0.054)	< 0.001	0.234 (0.099, 0.369)	< 0.001	0.004 (-0.012, 0.020)	0.606
ELSA	≥ 1 room per person	-0.056 (-0.130, 0.019)	0.142	0.265 (-0.279, 0.810)	0.339	-0.010 (-0.084, 0.065)	0.797
ELSA	Heating available	-0.072 (-0.098, -0.046)	< 0.001	-0.190 (-0.378, -0.002)	0.047	0.007 (-0.020, 0.034)	0.621
ELSA	Mobile phone	-0.060 (-0.102, -0.018)	0.005	0.366 (0.081, 0.650)	0.012	-0.005 (-0.047, 0.037)	0.814
ELSA	Computer	-0.075 (-0.083, -0.067)	< 0.001	0.083 (-0.009, 0.176)	0.077	0.018 (0.007, 0.029)	0.001
ELSA	Any housing problem	-0.065 (-0.072, -0.059)	< 0.001	-0.328 (-0.418, -0.237)	< 0.001	-0.001 (-0.013, 0.011)	0.879
ELSA	Too cold in winter	-0.065 (-0.071, -0.060)	< 0.001	-0.422 (-0.667, -0.177)	< 0.001	-0.007 (-0.040, 0.025)	0.654
ELSA	Any home adaptation	-0.064 (-0.071, -0.058)	< 0.001	-0.638 (-0.730, -0.546)	< 0.001	-0.005 (-0.018, 0.008)	0.436

Growth-curve linear mixed-effects models with time in years since baseline (continuous). Each housing indicator was entered in a separate model with a housing×time interaction. “Annual change in IC in reference group” represents the estimated yearly change in intrinsic capacity (IC) for the reference category of each housing characteristic. “Baseline difference (Yes–No)” represents the estimated difference in IC level between participants with and without the housing characteristic. “Slope difference per year (Yes–No)” represents the housing × time interaction term and indicates whether the annual rate of IC change differs by housing characteristic. Models adjusted for baseline age, sex, marital status, education, household wealth tertiles, and number of chronic diseases (missing values retained using a “Missing/Unknown” category). Participant-level random intercepts and random slopes for time were included (1 + t | id). Estimates are β (95% CI) and *p*-values for the No reference group time slope, baseline Yes–No difference, and Yes–No slope difference

In CHARLS, the annual change in IC in the reference group ranged from − 0.101 to − 0.117 per year. Significant baseline IC differences were observed for several housing characteristics. Higher baseline IC was associated with at least one room per person (0.188, *p* < 0.001), heating available (0.326, *p* < 0.001), electricity supply (0.187, *p* < 0.001), running water supply (0.378, *p* < 0.001), gas supply (0.331, *p* < 0.001), telephone connection (0.190, *p* < 0.001), internet connection (0.471, *p* < 0.001), indoor toilet (0.192, *p* < 0.001), and indoor toilet with seat (0.466, *p* < 0.001). Lower baseline IC was observed for home ownership (− 0.203, *p* < 0.001), elevator (− 0.393, *p* < 0.001), and handicapped/access features (− 0.210, *p* < 0.001). No housing × time interaction was clearly observed in CHARLS.

In ELSA, the annual change in IC in the reference group was also generally negative. It ranged from − 0.060 to − 0.075 per year for most housing indicators and was statistically significant for all except at least one room per person (− 0.056, *p* = 0.142). Significant baseline IC differences were observed for home ownership (0.234, *p* < 0.001), heating available (− 0.190, *p* = 0.047), mobile phone (0.366, *p* = 0.012), any housing problem (− 0.328, *p* < 0.001), too cold in winter (− 0.422, *p* < 0.001), and any home adaptation (− 0.638, *p* < 0.001). The only significant housing × time interaction in the primary ELSA models was for computer ownership, which was associated with a more favourable IC trajectory over follow-up (0.018, *p* = 0.001).

Sensitivity analyses are presented separately in Supplementary Tables S4–S6. Supplementary Table S4 compares baseline characteristics of complete and incomplete cases to assess attrition. Supplementary Table S5 presents inverse-probability-weighted mixed-model results, and Supplementary Table S6 presents post-baseline mixed models adjusted for baseline IC and baseline covariates. Results from sensitivity analyses showed evidence of selective attrition in both cohorts (Supplementary Table S4). Incomplete cases had lower baseline IC and were older in both CHARLS and ELSA, and also differed in several socio-demographic and housing characteristics. In inverse-probability-weighted mixed-model sensitivity analyses (Supplementary Table S5), some associations differed from the unweighted analyses. Specifically, inverse-probability-weighted models showed significant housing × time interaction terms in CHARLS for heating (0.112, *p* = 0.025), coal / natural gas supply (0.092, *p* = 0.022), internet connection (0.087, *p* = 0.033), and indoor toilet with seat (0.109, *p* = 0.004). In ELSA, most housing × time terms remained non-significant; the only significant slope difference was for phone ownership, which was associated with a less favourable trajectory (− 0.071, *p* = 0.033). In sensitivity analyses restricted to post-baseline observations and adjusted for baseline IC and baseline covariates (Supplementary Table S6), the overall pattern of weak housing-related trajectory effects remained largely unchanged. In CHARLS, housing × time interaction was also not clearly observed. In ELSA, the positive trajectory association observed for computer ownership in the primary models attenuated to the null after baseline-IC adjustment, whereas home adaptation became significantly associated with faster decline in IC over follow-up.

## Discussion

Using data from two nationally representative cohorts across different settings, this study showed that several housing characteristics were associated with baseline IC differences, whereas evidence for differential IC trajectories was limited. In the primary growth-curve models, living space, communication access, and several utility-related housing indicators were associated with higher baseline IC in CHARLS, while in ELSA a smaller number of housing indicators were associated with baseline IC level. In sensitivity analyses, most housing × time interaction terms remained weak. After adjustment for baseline IC, the overall pattern of limited trajectory differences remained largely unchanged, while many baseline differences comparing with and without the housing characteristics concerned were attenuated. These findings suggest that baseline functional and social differences explain an important part of the observed housing–IC associations.

A further consideration is that participants were already relatively old at baseline in both cohorts, and many of the housing characteristics examined are likely to reflect long-term living conditions accumulated over much of the life course. In that context, baseline IC may already embody the cumulative influence of prolonged exposure to these housing environments. By contrast, the relatively short period of follow-up and the limited within-person change in housing conditions during later life may make it less likely that substantial differences in subsequent IC trajectories would be detected over only a few additional years of observation.

This study observed a consistent positive association between sufficient living space and IC across settings. These findings align with previous research using CHARLS data, which demonstrated that individuals with at least one room per household member were less likely to experience declines in healthy ageing score, a composite measure including six physiological domains such as cognitive and kidney function [29]. The finding on the importance of adequate living space is consistent with the WHO’s recognition of household crowding as a significant health threat [7]. Several mechanisms may explain the observed associations between living space and IC. Household overcrowding often results in insufficient personal space, lack of privacy, and poor rest conditions, all of which might contribute to stress and poorer mental health [13, 14]. Adequate living space may also facilitate social interaction and home-based activity, both of which may support psychological well-being and locomotion [15]. In addition, overcrowding may increase the risk of infectious diseases due to close contact with others and poor ventilation, potentially leading to illness and functional decline [13]. Together, these mechanisms suggest that sufficient living space may support several dimensions of IC simultaneously.

Cross-country differences in the estimated associations should be interpreted cautiously because the same housing indicator may not represent the same underlying condition in England and China. For example, heating was almost universal in ELSA (95.0%) but uncommon in CHARLS (9.2%), suggesting that ‘heating’ in the two cohorts may capture different aspects of climate, housing quality, infrastructure, and material living conditions. Similarly, at least one room per person was nearly universal in ELSA but much less common in CHARLS, meaning that this variable may distinguish relative advantage in CHARLS more strongly than in ELSA. More generally, differences in infrastructure, climate, housing systems, and socioeconomic development are likely to influence both measurement and effect size, and the findings should therefore be interpreted as context-specific rather than as direct cross-country contrasts.

In this study, we have observed that some adaptation-related measures in CHARLS, such as indoor toilets, were associated with higher baseline IC, whereas others, such as elevators, were associated with lower baseline IC. However, most housing × time interaction terms were weak, and many of these baseline differences were attenuated after adjustment for baseline IC. These patterns suggest that adaptation-related variables may capture different aspects of the housing environment and may also reflect pre-existing functional limitation or selective need. For example, indoor toilets may indicate better sanitation, safer daily functioning, and greater independence, whereas elevators or other adaptations may be more common among participants whose functional difficulties had already emerged [11, 17, 18]. Accordingly, negative associations for some adaptation-related variables should not be interpreted as evidence that these features are harmful; rather, they may reflect underlying reverse causality, selective need, or greater pre-existing functional impairment among those who require them [30]. This interpretation is also consistent with the baseline-IC adjusted sensitivity analyses, in which home adaptation in ELSA was associated with a less favourable trajectory after accounting for initial IC.

We observed a consistent relationship between ownership of phone and better IC in both settings. While phone ownership can be a proxy for socioeconomic status, its significance after controlling for wealth suggests a more direct role. This aligns with extensive research on the detrimental health effects of social isolation. A phone could be an important tool to maintain social connection, which is essential for maintaining mental health (psychological domain) and accessing emergency help and telehealth services (vitality and locomotor domains). This is especially important when the older people have limited digital literacy to get connected to family, friends and essential services using online means [19]. Nonetheless, we wish to note that the prevalence and social meaning of communication-related resources also differed substantially between cohorts, so these indicators should be interpreted in relation to each country’s broader technological and social context rather than as equivalent exposures.

The findings from the current study aligned with the WHO Housing and Health Guideline by recognizing housing as a major entry point for primary prevention [7]. We added to the evidence that housing characteristics could be important for promoting different domains of IC. These pathways are unlikely to be domain-specific in isolation. Rather, housing characteristics may simultaneously shape several dimensions of intrinsic capacity, for example by affecting physical safety, social interaction, stress, and access to basic resources. However, the policy implications of these findings are likely to differ by national context. In CHARLS, where access to several basic utilities and communication resources was less common and some of these indicators were associated with more favourable IC levels and/or trajectories, the findings support policies that improve access to essential housing infrastructure, including heating, water, gas, sanitation, and communication access. In ELSA, where many basic housing resources were already near-universal, the findings are less consistent with broad infrastructure expansion and instead suggest the importance of identifying older adults living with housing problems or adaptation needs as groups potentially requiring closer functional and social support. More generally, the results support the integration of housing considerations into age-friendly and healthy-ageing policy, but the specific priorities are likely to differ between settings with different levels of infrastructure, social provision, and economic development [31]. Implementation should therefore be considered in light of local barriers and facilitators. In settings where basic housing infrastructure remains uneven, material improvements may be a more immediate priority, whereas in settings with higher baseline housing standards, the policy focus may shift toward accessibility, maintenance, identification of risk, and coordination between housing and health services [32].

Notwithstanding the strengths of our study, including the use of large, longitudinal, and nationally representative datasets from two diverse countries, some limitations exist. First, the measurement of IC relied on available data items across the five domains of IC that were harmonizable in both cohorts. A summation of domain score was used to derive the IC score. Although there is no consensus yet for the definitive way of defining IC, previous studies showed that this IC derived from this method was consistently related to different health outcomes in both cohorts [33, 34]. Second, although the longitudinal design strengthens temporal ordering relative to a purely cross-sectional analysis, the housing indicators were largely time-invariant and most housing × time associations were weak. We therefore cannot infer strong causal effects of housing conditions on within-person IC change, and the findings should be interpreted primarily as evidence of associations between housing-related environmental conditions, baseline IC differences, and repeated IC observations over follow-up. Third, selective attrition was an important issue in both cohorts. Participants with incomplete IC follow-up had lower baseline IC and were older than those retained in the main analyses, and they also differed in several socioeconomic and housing characteristics. Attrition-weighted sensitivity analyses suggested that selective dropout may have attenuated some associations, particularly in CHARLS, although the overall pattern of results remained similar. Fourth, direct comparison of housing indicators across ELSA and CHARLS could be constrained by major contextual differences in prevalence, infrastructure, and likely measurement meaning. Accordingly, the cross-country findings should be interpreted primarily as evidence of context-specific associations rather than as strict effect-size comparisons between England and China. However, the inclusion of two contrasting settings remains a strength of the study because it allows both common and context-specific patterns to be identified [20].

In conclusion, this large-scale, longitudinal, and cross-national study shows that housing characteristics were associated primarily with baseline IC differences, with limited evidence of differential IC trajectories over follow-up. Adequate living space and communication access were consistently related to better IC levels, while utility-related associations were more evident in CHARLS than in ELSA. The findings highlight both potentially common pathways, such as the importance of personal space and communication access, and context-specific housing features linked to infrastructure and development. Overall, the results support the relevance of housing within healthy-ageing policy, while also indicating that the meaning and implications of specific housing characteristics differ across settings and should be interpreted cautiously.

## Supplementary Information

Supplementary Material 1

## Data Availability

This study used publicly available data, which could be accessed through the Gateway to Global Aging Data at https://g2aging.org/.
