# Supplementary material for: Living space, utilities, and communication access as determinants of intrinsic capacity: longitudinal findings from England and China
Source: BMC Geriatr. 2026 Apr 24;26:811. doi: 10.1186/s12877-026-07549-w (PMC13245027; doi:10.1186/s12877-026-07549-w)
Supplement: Supplementary file 1 — Supplementary Material 1 [file 12877_2026_7549_MOESM1_ESM.docx]

**Supplementary Table S1. Measurements of IC**

| **Domains** | **Variables** | **Score** | **Measurements in ELSA** | **Measurements in CHARLS** |
| --- | --- | --- | --- | --- |
| Cognition | Memory |  | Immediate recall and delayed recall (stratified by age groups to define the cut-off point)^1^ | |
|  |  | 0 | · ≤ (Mean - SD) | |
|  |  | 1 | · > (Mean - SD) | |
|  | Orientation |  | Recalling year, month, day, and day of the week (stratified by age groups to define the cut-off point) | |
|  |  | 0 | · ≤ (Mean - SD) | |
|  |  | 1 | · > (Mean - SD) | |
| Psychological health | Depression |  | CES-D (8 items)^2^ | CES-D (10 items)^3^ |
|  |  | 0 | · 4–8 | · 20–30 |
|  |  | 1 | · 2–3 | · 10–19 |
|  |  | 2 | · 0–1 | · 0–9 |
| Sensory function | Vision |  | Self-rated vision | Self-rated close/distant vision |
|  |  | 0 | · Poor or blind | · Poor or blind |
|  |  | 1 | · Excellent, very good, good, or fair | · Excellent, very good, good, or fair |
|  | Hearing |  | Self-rated hearing | |
|  |  | 0 | · Poor | |
|  |  | 1 | · Excellent, very good, good, or fair | |
| Locomotion | Walking difficulty |  | Self-rated difficulty with walking 100 yards/meters/one block | |
|  |  | 0 | · Yes | |
|  |  | 1 | · No | |
|  | Chair stand difficulty |  | Self-rated difficulty with getting up from a chair after sitting for long periods | |
|  |  | 0 | · Yes | |
|  |  | 1 | · No | |
| Vitality | Grip strength |  | The highest value was considered for the analysis^4^ | The highest value was considered for the analysis^5^ |
|  |  | 0 | · Male: < 30 kg; Female: < 20 kg | · Male: < 28 kg; Female: < 18 kg |
|  |  | 1 | · Male: ≥ 30 kg; Female: ≥ 20 kg | · Male: ≥ 28 kg; Female: ≥ 18 kg |
|  | BMI | 0 | · Underweight: < 18.5 | · Underweight: < 18.5 |
|  |  |  | · Obese: ≥ 30 | · Obese: ≥ 25 |
|  |  | 1 | · Normal weight: 18.5–24.9 | · Normal weight: 18.5–23.9 |
|  |  |  | · Overweight: 25–29.9 | · Overweight: 24–24.9 |

Note:

1. Petersen RC, Roberts RO, Knopman DS, et al. Prevalence of mild cognitive impairment is higher in men: The Mayo Clinic Study of Aging. Neurology 2010; 75: 889-897

2. White J, Zaninotto P, Walters K, et al. Severity of depressive symptoms as a predictor of mortality: the English longitudinal study of ageing. Psychol Med 2015; 45: 2771-2779

3. Zeng J, Lai X, Wang S, et al. Association of depressive symptoms with chronic liver disease among middle-aged and older adults in China. Front Psychiatry 2023; 14: 1273754

4. Cruz-Jentoft AJ, Baeyens JP, Bauer JM, et al. Sarcopenia: European consensus on definition and diagnosis: Report of the European Working Group on Sarcopenia in Older People. Age Ageing 2010; 39: 412-423

5. Chen LK, Woo J, Assantachai P, et al. Asian Working Group for Sarcopenia: 2019 Consensus Update on Sarcopenia Diagnosis and Treatment. J Am Med Dir Assoc 2020; 21: 300-307.e2

**Supplementary Table S2. Measurements of housing conditions in ELSA**

| **Housing conditions** | **Measurements in ELSA** |
| --- | --- |
| Home ownership | The respondent owns the accommodation outright/buys it with the help of a mortgage or loan |
| Average number of rooms per person ≥1 | The number of bedrooms divided by the number of people living in the household ≥1 |
| Heating | The respondent has any form of central heating in the accommodation |
| Durables owned | |
| Television | The respondent has any of the following items:  1) Television  2) Video recorder  3) CD player  4) Deep freeze or fridge freezer (exclude fridge only)  5) Washing machine  6) Tumble Dryer / Washer - Dryer  7) Dish washer  8) Microwave oven  9) Computer  10) On-line-digital / Satellite / Cable Television  11) Phone (landline)  12) DVD player |
| Video recorder |  |
| CD player |  |
| Deep freeze / Fridge freezer |  |
| Washing machine |  |
| Dish washer |  |
| Microwave oven |  |
| Computer |  |
| On-line-digital/Satellite/Cable Television |  |
| Phone |  |
| DVD player |  |
| Adaptions in property | |
| Widened doorways | The respondent’s home has any of the features to assist people who have physical impairments or health problems:  1) Widened doorways or hallways;  2) Ramps or street level entrances;  3) Hand rails;  4) Automatic or easy open doors;  5) Accessible parking or drop off site;  6) Bathroom modifications;  7) Kitchen modifications;  8) Lift;  9) Chair lift or stair glide;  10) Alerting devices, such as button alarms. |
| Ramps |  |
| Hand rails |  |
| Automatic doors |  |
| Accessible parking site |  |
| Bathroom modifications |  |
| Kitchen modifications |  |
| Lift |  |
| Chair lift or stair glide |  |
| Alerting devices |  |
| Problems in accommodation | |
| Shortage of space | The respondent’s accommodation has any of these problems:  1) Shortage of space;  2) Noise from neighbors;  3) Other street noise, such as traffic, businesses, factories;  4) Too dark, not enough light;  5) Pollution, grime or other environmental problems caused by traffic or industry;  6) Rising damp in floors and walls;  7) Water getting in from roof, gutters or windows;  8) Bad condensation problem;  9) Problems with electrical wiring or plumbing;  10) General rot and decay;  11) Problems with insects, mice or rats;  12) Too cold in winter. |
| Noise from neighbours |  |
| Other street noise |  |
| Too dark |  |
| Environmental problems |  |
| Rising damp |  |
| Water leaking in |  |
| Bad condensation problems |  |
| Problems with electrical wiring or plumbing |  |
| Rot and decay |  |
| Problems with insects, mice or rats |  |
| Too cold in winter |  |

**Supplementary Table S3. Measurements of housing conditions in CHARLS**

| **Housing conditions** | **Measurements in CHARLS** |
| --- | --- |
| Average number of rooms per person ≥1 | The number of bedrooms divided by the number of people living in the household ≥1 |
| Heating | The respondent's residence has heating |
| Electricity supply | The respondent's residence has electricity |
| Running water supply | The respondent's residence has running water |
| Coal/natural gas supply | The respondent's residence has coal gas or natural gas supply |
| Telephone connection | The respondent's residence has a telephone connection |
| Internet connection | The respondent's residence has broad-band internet connection |
| Elevator | The respondent's residence has an elevator, or the respondent live on the first floor |
| Handicapped facilities | The respondent's residence has any handicapped facilities (e.g., non-stair ramp) |
| Indoor toilet | The respondent's residence has at least one toilet |
| Indoor toilet with a seat | The respondent's residence has at least one toilet with a seat |

**Supplementary Table S4 – Baseline characteristics of complete and incomplete cases in CHARLS and ELSA**

| **Variable** | **Category** | **Incomplete cases** | **Complete cases** | **P value** |
| --- | --- | --- | --- | --- |
| **CHARLS** |  |  |  |  |
| Baseline intrinsic capacity |  | 7.87 (1.62) | 8.22 (1.46) | <0.001 |
| Age |  | 67.67 (6.21) | 66.50 (5.59) | <0.001 |
| Male | Yes | 48.8% | 57.1% | <0.001 |
| Married / partnered | Yes | 79.3% | 82.5% | 0.003 |
| Education | Primary | 93.3% | 92.9% | 0.126 |
|  | Secondary | 5.0% | 4.5% |  |
|  | Tertiary | 1.8% | 2.5% |  |
| Household wealth tertile | Low | 28.2% | 27.3% | <0.001 |
|  | Middle | 22.7% | 24.3% |  |
|  | High | 20.8% | 25.7% |  |
|  | Missing / unknown | 28.3% | 22.7% |  |
| Number of chronic conditions | 0 | 49.8% | 47.4% | 0.016 |
|  | 1 | 30.8% | 33.8% |  |
|  | ≥2 | 17.5% | 17.6% |  |
|  | Missing / unknown | 1.9% | 1.2% |  |
| Home ownership | Yes | 81.5% | 80.5% | 0.366 |
| At least 1 room per person | Yes | 53.4% | 54.8% | 0.298 |
| Heating | Yes | 8.9% | 9.5% | 0.423 |
| Electricity supply | Yes | 81.5% | 81.7% | 0.870 |
| Running water | Yes | 59.1% | 63.1% | 0.002 |
| Coal / natural gas supply | Yes | 12.0% | 12.2% | 0.861 |
| Telephone connection | Yes | 49.4% | 53.0% | 0.009 |
| Internet connection | Yes | 10.2% | 10.5% | 0.731 |
| Elevator | Yes | 82.7% | 81.0% | 0.101 |
| Handicapped facilities | Yes | 82.6% | 83.1% | 0.611 |
| Indoor toilet | Yes | 71.7% | 72.8% | 0.376 |
| Indoor toilet with seat | Yes | 16.1% | 14.6% | 0.124 |
|  |  |  |  |  |
| **ELSA** |  |  |  |  |
| Baseline intrinsic capacity |  | 7.93 (1.73) | 8.23 (1.60) | <0.001 |
| Age |  | 71.68 (7.66) | 70.25 (7.48) | <0.001 |
| Male | Yes | 40.6% | 47.1% | <0.001 |
| Married / partnered | Yes | 60.9% | 67.7% | <0.001 |
| Education | Primary | 44.1% | 43.1% | 0.889 |
|  | Secondary | 36.0% | 37.0% |  |
|  | Tertiary | 10.3% | 10.3% |  |
|  | Missing / unknown | 9.6% | 9.5% |  |
| Household wealth tertile | Low | 34.6% | 29.8% | <0.001 |
|  | Middle | 31.4% | 33.7% |  |
|  | High | 32.5% | 35.6% |  |
|  | Missing / unknown | 1.5% | 0.8% |  |
| Number of chronic conditions | 0 | 36.0% | 37.7% | 0.403 |
|  | 1 | 38.9% | 37.1% |  |
|  | ≥2 | 25.1% | 25.2% |  |
| Home ownership | Yes | 80.1% | 83.3% | 0.005 |
| At least 1 room per person | Yes | 99.4% | 99.5% | 0.829 |
| Heating | Yes | 94.6% | 95.2% | 0.294 |
| Phone ownership | Yes | 97.8% | 97.9% | 0.920 |
| Computer ownership | Yes | 42.7% | 45.8% | 0.031 |
| Any housing problem | Yes | 27.8% | 27.1% | 0.621 |
| Too cold in winter | Yes | 2.6% | 2.9% | 0.442 |
| Any home adaptation | Yes | 31.0% | 26.7% | 0.001 |

Complete follow-up indicates non-missing IC values at all observed follow-up waves, whereas incomplete follow-up indicates missing IC at one or more observed follow-up waves. Values are mean (SD) for continuous variables, % yes for binary variables, and category-specific percentages for multi-category variables. P values are from Welch two-sample t-tests for continuous variables and chi-square tests (or Fisher’s exact tests where appropriate) for categorical variables.

**Supplementary Table S5 – Inverse-probability-weighted mixed-model sensitivity analyses of housing characteristics and intrinsic capacity trajectories in CHARLS and ELSA**

| Cohort | Housing characteristic | Annual change in IC (reference group) | P (annual) | Baseline difference (Yes–No) | P (baseline) | Slope difference per year (Yes–No) | P (slope) |
| --- | --- | --- | --- | --- | --- | --- | --- |
| CHARLS | Home ownership | -0.150*** (0.036) | <0.001 | -0.201 (0.129) | 0.120 | -0.003 (0.039) | 0.936 |
| CHARLS | At least 1 room per person | -0.139*** (0.022) | <0.001 | 0.284** (0.094) | 0.003 | -0.027 (0.029) | 0.354 |
| CHARLS | Heating | -0.160*** (0.015) | <0.001 | 0.123 (0.165) | 0.458 | 0.112* (0.050) | 0.025 |
| CHARLS | Electricity supply | -0.152*** (0.034) | <0.001 | 0.209 (0.122) | 0.088 | -0.001 (0.037) | 0.982 |
| CHARLS | Running water | -0.169*** (0.024) | <0.001 | 0.265** (0.098) | 0.007 | 0.029 (0.030) | 0.333 |
| CHARLS | Coal / natural gas supply | -0.162*** (0.016) | <0.001 | 0.130 (0.131) | 0.322 | 0.092* (0.040) | 0.022 |
| CHARLS | Telephone connection | -0.167*** (0.022) | <0.001 | 0.085 (0.095) | 0.372 | 0.028 (0.029) | 0.332 |
| CHARLS | Internet connection | -0.160*** (0.015) | <0.001 | 0.285* (0.135) | 0.035 | 0.087* (0.041) | 0.033 |
| CHARLS | Elevator | -0.138*** (0.032) | <0.001 | -0.360** (0.115) | 0.002 | -0.017 (0.036) | 0.629 |
| CHARLS | Handicapped facilities | -0.174*** (0.033) | <0.001 | -0.259* (0.118) | 0.029 | 0.021 (0.036) | 0.561 |
| CHARLS | Indoor toilet | -0.162*** (0.028) | <0.001 | 0.126 (0.108) | 0.242 | 0.013 (0.033) | 0.690 |
| CHARLS | Indoor toilet with seat | -0.168*** (0.016) | <0.001 | 0.143 (0.127) | 0.260 | 0.109** (0.038) | 0.004 |
| ELSA | Home ownership | -0.070*** (0.014) | <0.001 | 0.317* (0.131) | 0.016 | -0.008 (0.014) | 0.554 |
| ELSA | At least 1 room per person | -0.072 (0.053) | 0.174 | 0.210 (0.426) | 0.621 | -0.004 (0.053) | 0.936 |
| ELSA | Heating | -0.067** (0.021) | 0.002 | -0.041 (0.177) | 0.816 | -0.009 (0.022) | 0.662 |
| ELSA | Phone ownership | -0.007 (0.033) | 0.842 | 0.833** (0.302) | 0.006 | -0.071* (0.033) | 0.033 |
| ELSA | Computer ownership | -0.079*** (0.007) | <0.001 | 0.130 (0.080) | 0.106 | 0.005 (0.009) | 0.597 |
| ELSA | Any housing problem | -0.077*** (0.005) | <0.001 | -0.362*** (0.087) | <0.001 | 0.002 (0.010) | 0.835 |
| ELSA | Too cold in winter | -0.075*** (0.005) | <0.001 | -0.276 (0.236) | 0.242 | -0.026 (0.028) | 0.363 |
| ELSA | Any home adaptation | -0.072*** (0.005) | <0.001 | -0.435*** (0.095) | <0.001 | -0.017 (0.011) | 0.126 |

Values are regression coefficients with standard errors in parentheses. “Annual change in IC (reference group)” represents the estimated yearly change in intrinsic capacity (IC) for the reference category of each housing characteristic. “Baseline difference (Yes–No)” represents the estimated difference in IC level between participants with and without the housing characteristic. “Slope difference per year (Yes–No)” represents the housing × time interaction term and indicates whether the annual rate of IC change differs by housing characteristic. As a sensitivity analysis for selective attrition, models were refitted using inverse-probability-of-inclusion weights estimated from baseline logistic regression models of analytic-sample inclusion. Weighted mixed models were fitted using WeMix in R programming, with unit weights at the repeated-measure level and attrition weights at the participant level. Positive slope-difference estimates indicate slower IC decline (or more favourable IC trajectories) among participants with the housing characteristic, whereas negative estimates indicate faster decline. P values refer to Wald tests of the corresponding coefficients. Asterisks denote statistical significance (p<0.05, *p<0.01, **p<0.001).

**Supplementary Table S6. Baseline-IC adjusted mixed-model sensitivity analyses of housing characteristics and subsequent intrinsic capacity trajectories in CHARLS and ELSA**

| Cohort | Housing characteristic | Annual change in IC (reference group) | P (annual) | Baseline difference (Yes–No) | P (baseline) | Slope difference per year (Yes–No) | P (slope) |
| --- | --- | --- | --- | --- | --- | --- | --- |
| CHARLS | Home ownership | -0.118** (0.037) | 0.002 | 0.023 (0.139) | 0.868 | -0.025 (0.042) | 0.548 |
| CHARLS | At least 1 room per person | -0.117*** (0.024) | <0.001 | 0.286** (0.107) | 0.008 | -0.041 (0.032) | 0.200 |
| CHARLS | Heating | -0.144*** (0.017) | <0.001 | 0.034 (0.194) | 0.859 | 0.094 (0.060) | 0.115 |
| CHARLS | Electricity supply | -0.118** (0.037) | 0.002 | 0.222 (0.138) | 0.109 | -0.024 (0.041) | 0.555 |
| CHARLS | Running water | -0.127*** (0.026) | <0.001 | 0.156 (0.111) | 0.161 | -0.017 (0.033) | 0.608 |
| CHARLS | Coal / natural gas supply | -0.142*** (0.017) | <0.001 | 0.128 (0.172) | 0.459 | 0.044 (0.053) | 0.398 |
| CHARLS | Telephone connection | -0.148*** (0.024) | <0.001 | -0.035 (0.108) | 0.745 | 0.020 (0.032) | 0.544 |
| CHARLS | Internet connection | -0.147*** (0.017) | <0.001 | -0.020 (0.182) | 0.913 | 0.108 (0.056) | 0.052 |
| CHARLS | Elevator | -0.126** (0.039) | 0.001 | -0.149 (0.142) | 0.294 | -0.013 (0.043) | 0.758 |
| CHARLS | Handicapped facilities | -0.151*** (0.042) | <0.001 | -0.161 (0.149) | 0.280 | 0.015 (0.045) | 0.743 |
| CHARLS | Indoor toilet | -0.133*** (0.031) | <0.001 | 0.040 (0.121) | 0.741 | -0.007 (0.036) | 0.844 |
| CHARLS | Indoor toilet with seat | -0.147*** (0.017) | <0.001 | 0.009 (0.161) | 0.953 | 0.064 (0.048) | 0.186 |
| ELSA | Home ownership | -0.089*** (0.014) | <0.001 | 0.078 (0.108) | 0.472 | 0.008 (0.015) | 0.584 |
| ELSA | At least 1 room per person | -0.085 (0.064) | 0.187 | 0.087 (0.453) | 0.848 | 0.004 (0.065) | 0.955 |
| ELSA | Heating | -0.075** (0.025) | 0.003 | 0.002 (0.170) | 0.991 | -0.007 (0.025) | 0.795 |
| ELSA | Phone ownership | -0.011 (0.040) | 0.782 | 0.766** (0.258) | 0.003 | -0.071 (0.040) | 0.075 |
| ELSA | Computer ownership | -0.087*** (0.008) | <0.001 | 0.031 (0.071) | 0.659 | 0.010 (0.010) | 0.319 |
| ELSA | Any housing problem | -0.080*** (0.006) | <0.001 | -0.143 (0.076) | 0.059 | -0.003 (0.011) | 0.784 |
| ELSA | Too cold in winter | -0.080*** (0.005) | <0.001 | 0.138 (0.197) | 0.486 | -0.036 (0.029) | 0.211 |
| ELSA | Any home adaptation | -0.075*** (0.006) | <0.001 | -0.009 (0.082) | 0.912 | -0.027* (0.012) | 0.024 |

Values are regression coefficients with standard errors in parentheses. “Annual change in IC (reference group)” represents the estimated yearly change in intrinsic capacity (IC) for the reference category of each housing characteristic. “Baseline difference (Yes–No)” represents the estimated difference in post-baseline IC level between participants with and without the housing characteristic, conditional on baseline IC and baseline covariates. “Slope difference per year (Yes–No)” represents the housing × time interaction term and indicates whether the annual rate of IC change differs by housing characteristic. As a sensitivity analysis addressing baseline functional differences and possible reverse causality, models were restricted to post-baseline observations and adjusted for baseline IC together with baseline covariates. Positive slope-difference estimates indicate slower IC decline (or more favourable IC trajectories) among participants with the housing characteristic, whereas negative estimates indicate faster decline. P values refer to Wald tests of the corresponding coefficients. Asterisks denote statistical significance (p<0.05, *p<0.01, **p<0.001).
